# Molecular Characterization of *Donacia provosti* (Coleoptera: Chrysomelidae) Larval Transcriptome by De Novo Assembly to Discover Genes Associated with Underwater Environmental Adaptations

**DOI:** 10.3390/insects12040281

**Published:** 2021-03-25

**Authors:** Haixia Zhan, Youssef Dewer, Cheng Qu, Shiyong Yang, Chen Luo, Liangjun Li, Fengqi Li

**Affiliations:** 1 Beijing Key Laboratory of Environment Friendly Management on Fruit Diseases and Pests in North China, Institute of Plant and Environment Protection, Beijing Academy of Agriculture and Forestry Sciences, Beijing 100097, China; zhanertu@gmail.com (H.Z.); qucheng@ipepbaafs.cn (C.Q.); luochen@baafs.net.cn (C.L.); 2 Bioassay Research Department, Central Agricultural Pesticide Laboratory, Agricultural Research Center, Dokki, Giza 12618, Egypt; dewer72@gmail.com; 3 School of Ecology and Environment, Anhui Normal University, Wuhu 241000, China; shiyan@ahnu.edu.cn; 4 School of Horticulture and Plant Protection, Yangzhou University, Yangzhou 225009, China

**Keywords:** *Donacia provosti*, aquatic lifestyle, transcriptome assembly, positive selection, adaptive evolution

## Abstract

**Simple Summary:**

*Donacia provosti* is one of the major pests of aquatic crops. It has been widely distributed in the world causing extensive damage to lotus and rice plants. The larvae generally live-in water; however, little is known about the evolution and molecular mechanisms underlying the adaptation. Here, we generated the first larval transcriptome of *D. provosti* in order to identify potential genetic mechanisms of aquatic adaptation. About 5036 orthologous clusters were identified among four species and 494 unique clusters were identified from *D. provosti* larvae including the visual perception. Moreover, 93 orthologous gene pairs were found evolving under positive selection. Our results also showed that 4 gene pairs out of the 93 gene pairs were associated with the “mTOR signaling pathway”, which are predicted to be involved in the molecular mechanism of *D. provosti* adaptation to the underwater environment. In the light of the increasing availability of transcriptomic information for beetle underwater habitat and evolutionary analyses, it is expected that this paper will provide us with some novel insights into aquatic adaptation in beetles and serves as a foundation for future studies aiming to identify candidate genes underlying the genetic basis of aquatic adaptation in beetles.

**Abstract:**

*Donacia provosti* (Fairmaire, 1885) is a major pest of aquatic crops. It has been widely distributed in the world causing extensive damage to lotus and rice plants. Changes in gene regulation may play an important role in adaptive evolution, particularly during adaptation to feeding and living habits. However, little is known about the evolution and molecular mechanisms underlying the adaptation of *D. provosti* to its lifestyle and living habits. To address this question, we generated the first larval transcriptome of *D. provosti*. A total of 20,692 unigenes were annotated from the seven public databases and around 18,536 protein-coding genes have been predicted from the analysis of *D. provosti* transcriptome. About 5036 orthologous cutlers were identified among four species and 494 unique clusters were identified from *D. provosti* larvae including the visual perception. Furthermore, to reveal the molecular difference between *D. provosti* and the Colorado potato beetle *Leptinotarsa decemlineata*, a comparison between CDS of the two beetles was conducted and 6627 orthologous gene pairs were identified. Based on the ratio of nonsynonymous and synonymous substitutions, 93 orthologous gene pairs were found evolving under positive selection. Interestingly, our results also show that there are 4 orthologous gene pairs of the 93 gene pairs were associated with the “mTOR signaling pathway”, which are predicted to be involved in the molecular mechanism of *D. provosti* adaptation to the underwater environment. This study will provide us with an important scientific basis for building effective prevention and control system of the aquatic leaf beetle *Donacia provosti.*

## 1. Introduction

*Donacia provosti* (Fairmaire, 1885) (Coleoptera: Chrysomelidae) (syn. *D. brevicollis* and *D. yuasai*) is a highly harmful pest of aquatic crops [1,2]. In 1885, it was recorded for the first time in Beijing, China by Fairmaire [3]. Nowadays, *D. provosti* populations are increasing and their geographical distribution appears to be widening in different regions around the world, e.g., in Russia (Primorsky, and Amur), Korea, Japan (Hokkaido, Honshu, Shikoku, and Kyushu), and China [4]. In China, this pest is distributed from the south in Hainan to the north in Heilongjiang, especially in Hubei and Jiangsu provinces [5].

*D. provosti* feeds mainly on crops of economic importance, particularly the lotus *Nelumbo nucifera* Gaertn and the rice *Oryza sativa* L. The adults chew up lotus leaves and create nicks and holes, or leave only the epidermis [2]. Interestingly, the larvae live on the bottom of the water and bore into tender stems and roots of lotus, causing damage making them unmarketable [6]. The damaged tender stems cause dark brown spots and the roots are prone to mold and rot; the growth of the above-ground part is also affected to a certain extent, among which the standing leaves are yellow and withered, and the flower buds are thin (Figures S1 and S2) [7]. In China, *D. provosti* caused 15–20% loss of lotus root in the 2000s [8]. In 2012, *D. provosti* broke out on thousands of acres of lotus fields in Yuanjiang, Hunan Province [9]. Recently, the damage of the lotus root industry by this beetle has become more and more serious, so that it has become a major pest of lotus root.

Currently, the management of *D. provosti* is mainly dependent on chemical insecticides [10]. However, chemical control is not only costly, but also has resulted in water contamination which causes considerable adverse health effects in human and livestock populations [11]. Consequently, there is an urgent demand for developing an environment-friendly insect-pest management strategy to better suppress this pest and to cope with pollutants to water bodies. Meanwhile, very little is known about the genetic basis of aquatic environment adaptations in *D. provosti*. Unlike *D. provosti*, the caddisfly *Stenopsyche tienmushanensis* egg, larval, and pupal stages are entirely aquatic. Adaptation of *S. tienmushanensis* larval silks to aquatic habitats was found to be associated with serine phosphorylation [12,13]. In fact, the genome information of caddisfly provided us with an important resource for understanding the molecular mechanism involved in the adaptation of *D. provosti* larvae to their lifestyle and living habits [14]. Understanding these adaptations will help explain how insects, in general, have evolved as one of the most successful and abundant classes of animals on the planet and how *D. provosti* larvae, in particular, have adapted to aquatic ecosystems.

In this study, we sequenced and analyzed the larval transcriptome of *D. provosti* for the first time. These data will help to identify the key genes and molecular mechanisms underlying environmental adaptations of *D. provosti* larvae to their aquatic habits. It may also provide basic knowledge for designing novel strategies to control *D. provosti* in the future.

## 2. Materials and Methods

### 2.1. Insect Materials and RNA Isolation

*D. provosti* larvae samples were collected in July 2019 from the lotus root *N. nucifera* located in the Sheyang Lake town, Baoying County, Yangzhou city, Jiangsu province (119.62 E, 33.32 N). The larvae samples length ranged from 8 mm to 12 mm. All *D. provosti* samples were freshly collected from the fields and frozen immediately at −80 °C before molecular analysis. Sequencing was performed on three independent biological replicates, and each replicate includes 5 individual larvae. The standard Trizol method was used to isolate total RNA, and the Agilent Bioanalyzer 2100 system (Agilent Technologies, Santa Clara, CA, USA) was used to evaluate the integrity of all RNA samples.

### 2.2. cDNA Library Preparation and Illumina Sequencing

After sample RNA quality was assessed, the eukaryotic mRNA was enriched with magnetic beads with Oligo (dT) using NEBNext^®^ Ultra™ RNA Library Prep Kit for Illumina^®^ (NEB, Ipswich, MA, USA). Subsequently, fragmentation buffer is added to break the mRNA into short fragments. Using mRNA as a template, one-strand cDNA is synthesized with six-base random primers (random hexamers), and then buffer, dNTPs, DNA polymerase I and RNase H were added to synthesize two-strand cDNA. Purify double-stranded cDNA with AMPure XP beads. The purified double-stranded cDNA was first repaired, A-tailed and connected to the sequencing adapter. In order to select cDNA fragments of preferentially 250–300 bp in length, the library fragments were purified with AMPure XP system (Beckman Coulter, Beverly, MA, USA). Then 3 µL USER Enzyme (NEB, Ipswich, MA, USA) was used with size-selected, adaptor-ligated cDNA at 37 °C for 15 min followed by 5 min at 95 °C before PCR. Then PCR was performed with Phusion High-Fidelity DNA polymerase, Universal PCR primers and Index (X) Primer. At last, PCR products were purified (AMPure XP system) and library quality was assessed on the Agilent Bioanalyzer 2100 system. Illumina sequencing was performed for all three *D. provosti* larvae RNA samples.

### 2.3. Bioinformatics Analysis

The raw reads were obtained through high-throughput sequencing, and then after removing the adapters, low-quality bases and N bases are filtered out to obtain clean reads. Using the paired-end splicing method of Trinity v2.4.0 [15] software, the clean reads were spliced to obtain the Transcript sequence. Corset v 1.05 [16] uses the number of reads and expression patterns of the transcripts on the alignment to perform hierarchical clustering of transcripts. After Corset hierarchical clustering, the longest Cluster sequence was obtained for subsequent analysis. In order to obtain comprehensive gene function information, we performed gene function annotations using BLAST with a cutoff E-value of <10^−5^ in seven major databases: Nr, Nt, Pfam [17], KOG/COG, Swiss-Prot, and KO and Gene Ontology (GO) [18].

### 2.4. Protein-Coding Sequence (CDS) Prediction

CDS prediction was divided into two steps: The first step, unigenes alignment was performed according to the priority order of NR protein library and Swissprot protein library. If the comparison was corrected, the open reading frame (ORF) coding frame information of the transcript was extracted from the comparison result, and the coding region sequence was translated into amino acid sequence according to the standard codon table (in the order of 5′- > 3′). The second step, for the sequences with no aligned on the NR or Swissprot protein library, or the sequences with no predicted results, ESTScan (version 3.0.3) software (Swiss Institute of Bioinformatics, Lausanne, Switzerland) was used to predict their ORF, so as to obtain the nucleic acid sequence and amino acid sequence encoded by this part of the unigenes.

### 2.5. Orthologous Cluster Analysis

A total protein orthologous of four species including three beetle pests (the Asian long-horned beetle *Anoplophora glabripennis*, the Colorado potato beetle *Leptinotarsa decemlineata*, and *D. provosti*) and an aquatic insect (the caddisfly *S. tienmushanensis*) were analyzed with OrthoVenn2 (https://orthovenn2.bioinfotoolkits.net/task/create) (accessed on 19 February 2021) using the E-value of 1e^−5^ and an inflation value of 1.5 [19]. The sequences of *A. glabripennis* proteins were extracted from genome sequences [20]. The sequences of *L. decemlineata* proteins were extracted from genome sequences (NCBI BioProject number PRJNA420356, NCBI Assembly number GCF_000500325.1), with GXF Sequences Extract tool and Batch Translate CDS to Protein in tool TBtools [20]. The sequences of *S. tienmushanensis* proteins were extracted from genome sequences (Stenopsyche.tienmushanensis.pep.fa file) [14].

### 2.6. Adaptive Evolution Analysis

The Ka/Ks ratio was used to evaluate the positive and negative selection between *D. provosti* and *L. decemlineata* [21]. The YN method of KaKs_software [22] was used to calculate the Ka/Ks ratio of each orthologous gene pair [23]. GO and KEGG enrichment of the orthologous gene pairs of Ka/Ks ratio above 1 were analyzed with KOBAS3.0 (http://kobas.cbi.pku.edu.cn/kobas3/genelist/) (accessed on 14 December 2020) [24].

## 3. Results

### 3.1. Transcriptome Sequencing and Assembly

Sequenced data were deposited in the NCBI Sequence Read Archive with BioProject ID number PRJNA682017. We assembled a total of 158,217,018 clean reads and 75,658 transcripts from the larval transcriptome (Table 1 and Table 2). The Q20 scores (the average quality value) were above 97.5% (Table 1). Moreover, a total length of 44,479,461 bp and 34,118 unigenes were generated from the assembled reads of the three replicates (Table 2). The mean length and N50 of these unigenes were 1304 and 2194 bp, respectively (Table 2). In addition, analysis of the size distribution showed 12,745 single unigenes that are more than 1000 bp in length.

### 3.2. Function Annotation

Among the total generated unigenes 20,692 were successfully annotated at least in one database, accounting for 60.64% of the total. The largest proportion of annotation in a single database was obtained for NR (52.99%) followed by PFAM (35.22%), and GO (35.22%) (Table 3).

**Figure 1:**
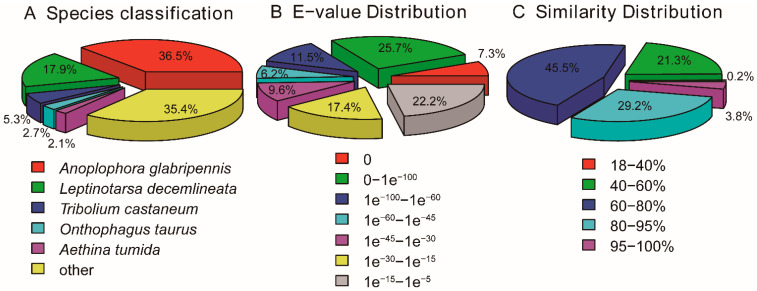
Pie charts showing distribution of the BLASTxmatches of *Donacia provosti* transcriptome unigenes against the NR database. (**A**) Species classification, (**B**) E-values distribution, and (**C**) Similarity distribution.

For main species distribution matched against the NR database, *D. provosti* unigenes have closely matched with sequences of *A. glabripennis* (36.6%) and *L. decemlineata* (17.9%). While, *D. provosti* unigenes have less than 5.3% matched with sequences of other species. (Figure 1A, Table S1). The E-value distributions showed that 60.4% of the annotated unigenes had significant homology (E-values < 1e^−30^) to other sequences in the NR database (Figure 1B). The similarity distributions showed that 78.5% of sequences had a similarity of more than 60%, while 21.5% sequences had a similarity ranging from 18% to 60% (Figure 1C).

We obtained a total of 67,134 functional annotations with GO functions, with most of the annotations belonging to biological processes (32,053), followed by cellular components (20,739), and molecular functions the least represented (14,284). Within the Biological process, cellular process, and metabolic process, and single-organism process were the most abundant. Cell and cell part terms were the most abundant categories among the Cellular component. For Molecular Function, unigenes were predominantly associated with binding and catalytic activity functions (Figure S3). In addition, within the binding, heterocyclic compound binding and ion binding were the most abundant.

The function prediction of all unigenes with KOG-based database showed that 6678 annotated putative proteins were assigned to 26 categories, mainly including (O) posttranslational modification, protein turnover and chaperones, (R) general function prediction only, (T) signal transduction mechanisms (Figure S4).

Following KEGG pathways analysis, we assigned a total of 6340 proteins to 229 KEGG pathways, with 1777 proteins (28.22%) being associated with metabolic pathways. The pathways involving the largest number of unigenes were signal transduction (731) in the environmental information processing, followed by translation (458) in the genetic information processing; biosynthesis of other secondary metabolism (5) was the least abundant (Figure S5).

### 3.3. Protein Coding Sequence (CDS) Prediction and Orthologous Analysis

**Figure 2:**
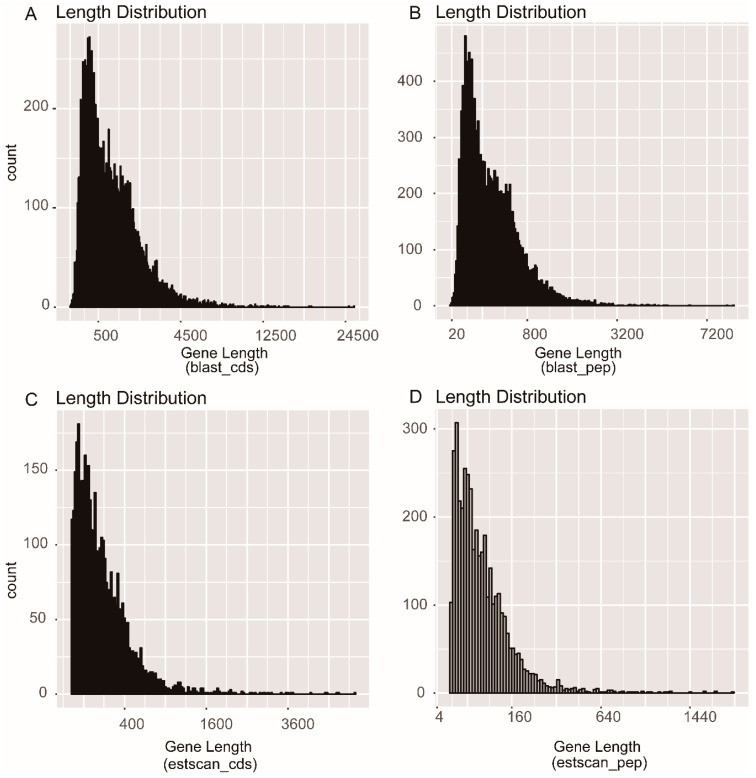
Coding sequence predictions of *Donacia provosti* transcriptome by BLASTx and ESTScan. Length distribution of (**A**) CDS using BLASTx (E-value < 1e^−5^), (**B**) proteins using BLASTx, (**C**) CDS predicted by ESTScan, and (**D**) proteins using ESTScan.

All 34,118 unigenes were compared with the protein databases, giving priority to NR and Swiss Prot. Using BLASTx, a total of 14,528 CDS were obtained from unigene sequences and translated into amino acid sequences (Figure 2A,B). Using ESTScan, we identified 4028 single-gene CDS that did not match the above protein database and translated them into amino acid sequences (Figure 2C,D).

### 3.4. Orthologous Cluster Analysis

**Figure 3:**
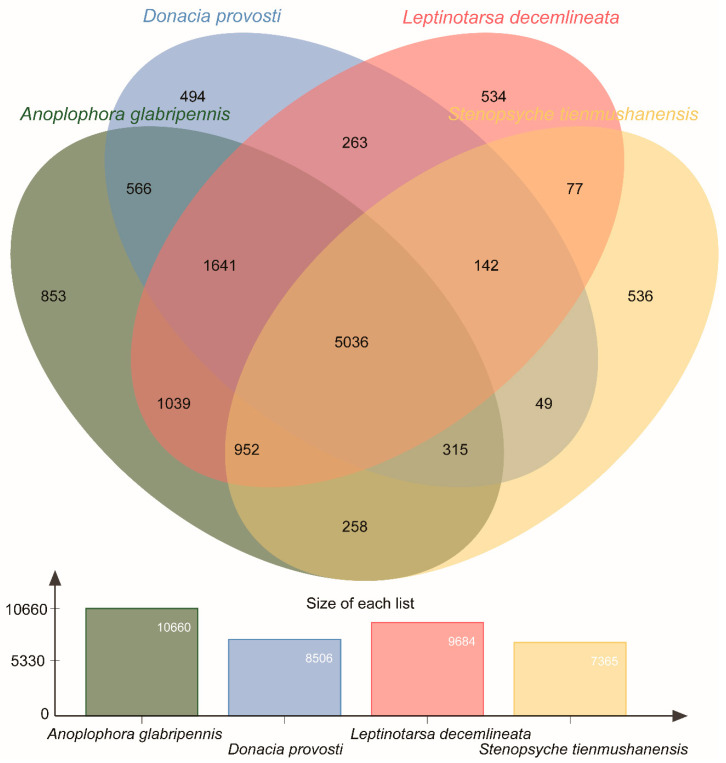
Venn diagram of gene homology among the four species annotations and number of gene homology in the four species annotations.

We identified 8506 clusters from 18,556 proteins in the *D. provosti* (Figure 3). A total of 5036 orthologous clusters were shared among all the four species (Figure 3), mainly including 44 counts of rRNA processing (GO: 0006364) and 28 counts of visual perception (GO:0007601) within the biological process by GO enriched (*p* < 0.05). Furthermore, 49 orthologous clusters were shared between *D. provosti* and *S. tienmushanensis* but not including *A. glabripennis* and *L. decemlineata* (Figure 3). GO enrichment analysis showed some orthologous clusters were significantly enriched for genes involved in nucleosome assembly (GO: 0006334) and DNA binding (GO: 0007601). However, 494 clusters were unique to *D. provosti* larval transcriptome data (Figure 3), mainly including the visual perception (GO:0007601), the DNA integration (GO: 0015074) within the biological process, and the RNA-directed DNA polymerase activity (GO: 0003964) within the molecular function by GO enriched (*p* < 0.05).

### 3.5. Adaptive Evolution Analysis

**Figure 4:**
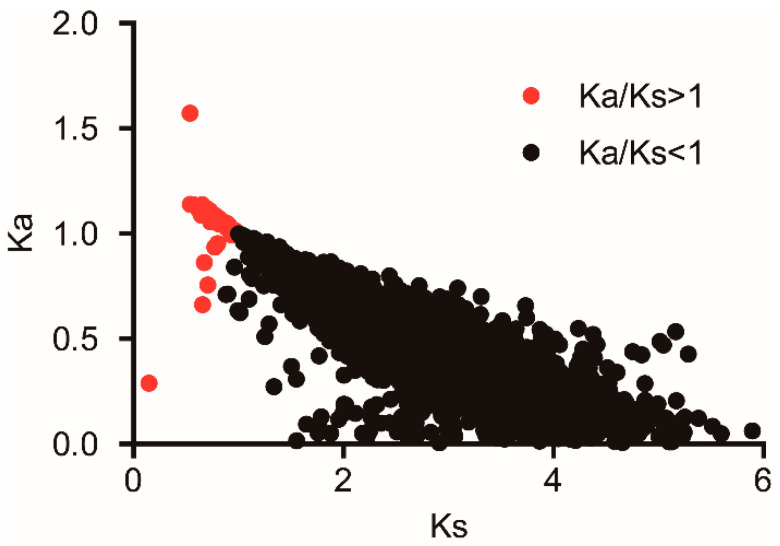
Distribution of Ka and Ks in 6627 orthologous gene pairs between *Donacia provosti* and *Leptinotarsa decemlineata*.

The maximum likelihood analysis of Ka and Ks was used to identify key genes reflecting adaptive evolution in the 6627 orthologous gene pairs between *D. provosti* and *L. decemlineata*. From these gene pairs, the mean values of Ka, Ks, and Ka/Ks ratio were 0.62, 2.21 and 0.34, respectively. The distribution of the Ka/Ks ratio showed that the majority of gene pairs (98.60%, 6534/6627) have Ka/Ks ratios less than 1, while 93 orthologous gene pairs between *D. provosti* and *L. decemlineata* were under positive selection (Figure 4; Table S2). GO annotation revealed that these 93 orthologous gene pairs were mainly involved in binding, membrane and metabolic process GO items (>10%). Interestingly, from 93 orthologous gene pairs, KEGG pathway analysis revealed four orthologous gene pairs, which were also present in the “mTOR signaling pathway” in the fruit fly *Drosophila melanogaster* (corrected *p* < 0.05, Figure S6). A number of orthologous gene pairs associated with “Spliceosome”, “Alanine, aspartate, and glutamate metabolism”, “Endocytosis”, “Pentose and glucuronate interconversions”, “Ribosome”, “Proteasome”, “RNA degradation”, “Biosynthesis of amino acids”, and “Ubiquinone and other terpenoid-quinone biosynthesis”, although not presenting a statistically significant over-representation at corrected *p* < 0.05 level, presented a statistically significant in *p* < 0.05 level.

## 4. Discussion

With the increasing development of high-throughput sequencing technology, research reports on the genome and transcriptome of non-model organisms are rising [25,26,27]. When the genome sequencing data are not available, transcriptome sequencing is an effective and accessible approach a to initiate comparative genomic analyses on non-model organisms, because they contain large number of protein-coding genes likely enriched for targets of natural selection. In this study, the first de novo transcriptome from *D. provosti* larvae was sequenced by the Illumina platform. We identified a total of 34,118 unigenes and 44,479,461 bp from *D. provosti* larval transcriptome. The Q20 scores were high (97.55%) and N50 was 2194 bp, indicating that the first de novo transcriptome is good and highly reliable. We also identified that a total of 60.64% annotated successfully at least in one database from the seven public databases and a maximum of 52.99% annotated successfully from the NCBI NR protein database. However, a large number of unigenes remained un-annotated, similarly to the results of previous studies [26,28], which may be due to the lack of *D. provosti* genome sequencing data and the limitations of the second-generation sequencing. These unigenes may be new genes or non-coding sequence that constitutes a unique transcript of the tested pest or the length of the spliced sequences could too short to obtain an aligned sequence.

We found that *D. provosti* unigenes closely matched with sequences of *A. glabripennis* and *L. decemlineata*, and 5036 orthologous clusters shared among all the four species were identified. However, we identified 494 clusters that were uniquely present to *D. provosti* larval transcriptome data compared with other species. The unique clusters could be novel proteins, evidence of unique horizontal gene transfer, or pseudogenes [29]. The unique genes are often associated with the individual’s unique phenotype, such as adaptability to a specific environment or unique disease resistance. The larval stages of *D. provosti* only live in the aquatic environment [6] among these three beetle species the remaining living on the land. These new genes should be important targets in future studies aiming at elucidating the genetic basis of adaptation to aquatic lifestyle of beetles. One of the most interesting findings is that 49 clusters of orthologous genes were shared between *D. provosti* and *S. tienmushanensis*, although the extreme differences in their orders and entire lifestyle [14]. GO enrichment analysis showed some orthologous clusters were significantly enriched for genes involved in the nucleosome assembly and DNA binding. These candidate genes are supposed to be involved in the adaptation of aquatic insects to their water environment. Therefore, this hypothesis needs to be further confirmed by population genomics in the future.

As previously described, the Ka/Ks ratio is an important index of molecular evolution. If the ratio of Ka/Ks is greater than one, it is considered under positive selection evolutionarily adaptive, and the higher ratio of Ka/Ks the stronger is the positive selection [21]. In our present evolutionary analysis between the two beetle species (*D. provosti* and *L. decemlineata*), most of the orthologous gene pairs had a Ka/Ks ratio less than one, and this indicates that the majority of orthologous genes were under strong purifying selection. However, we also detected these orthologous gene pairs undergoing adaptive evolution, among which 2 orthologous gene pairs had a Ka/Ks ratio greater than 2 and 91 orthologous gene pairs had a Ka/Ks ratio greater than 1, suggesting that these genes might play significant roles in adapting *D. provosti* larvae to survive underwater. However, further studies are still required to confirm that whether these gene pairs are involved in the environmental adaptation of *D. provosti* larvae to their aquatic living habits or not. Thus, our study established a starting point in understanding the evolution and molecular mechanisms of adaptation of *D. provosti* larvae to aquatic environment using molecular-based transcriptome analysis. Furthermore, four gene pairs between *D. provosti* and *L. decemlineata* associated with the “mTOR signaling pathway” under positive selection, suggesting that this process could play a role in the adaption of *D. provosti* larvae to underwater habitat. However further studies are still required to confirm that whether mTOR signaling pathway is involved in the environmental adaptation of *D. provosti* larvae to their aquatic living habits or not. Although the mTOR is a conserved serine/threonine protein kinase, the kinase mTOR integrates diverse environmental signals and translates these cues into appropriate cellular responses [30].

## 5. Conclusions

In this study, the larval transcriptome data of *D. provosti* were obtained and analyzed for the first time through high-throughput sequencing technology. This first large-scale transcriptomic dataset for *D. provosti* overall offers a valuable genetic resource for the study of gene function and could drive research on one of the most serious insect pests infesting aquatic crops. Meanwhile, the transcriptome resources produced by our study are useful to provide a foundation for further studies to identify candidate genes underlying adaptation to the aquatic lifestyle of beetles.

## Figures and Tables

**Table 1:** Output statistics from *D. provosti* larvae.

Replicates	Total Raw Reads	Total Clean Reads	Clean Bases (G)	Q20%	GC%
Larvae_1	50,681,752	49,714,298	7.46	97.53	39.95
Larvae_2	50,342,128	49,459,666	7.42	97.58	39.98
Larvae_3	60,752,838	59,043,054	8.86	97.54	40.25
All	161,776,718	158,217,018	23.74		

**Table 2:** Assembly statistics from *D. provosti* larvae.

De Novo Assembly	Total Number	Total Length (bp)	Mean Length (bp)	N50
Transcripts	75,658	117,120,238	1548	2722
Unigenes	34,118	44,794,61	1304	2194

**Table 3:** Summary of functional annotations of *D. provosti* unigenes.

Databases Annotation	Number of Unigenes	Percentage (%)
Annotated in NR	18,081	52.99
Annotated in NT	5685	16.66
Annotated in KO	6340	18.58
Annotated in SwissProt	10,040	29.42
Annotated in PFAM	12,036	35.27
Annotated in GO	12,036	35.27
Annotated in KOG	5999	17.58
Annotated in all Databases	2239	6.56
Annotated in at least one Database	20,692	60.64
Total Unigenes	34,118	100

## Data Availability

The raw data from Illumina sequencing were deposited in the NCBI Sequence Read Archive (SRA) database (BioProject ID PRJNA682017).

## Supplementary Materials

The following are available online at https://www.mdpi.com/2075-4450/12/4/281/s1, Figure S1: *Donacia provosti* larvae, Figure S2 *Donacia provosti* damage characteristics, Figure S3. GO Classification of *Donacia provosti* unigenes according to the categories of Biological process, Molecular function, and Cellular component. Figure S4. KOG annotations of *Donacia provosti* predicted proteins. A total of 6678 predicted proteins has a KOG classification among the 26 categories. Figure S5. KEGG annotation of *Donacia provosti* predicted proteins. A Cellular Processes, B Environmental Information Processing, C Genetic Information Processing, D Metabolism, E Organismal Systems. Figure S6 mTOR signaling pathway. Table S1 List of species with number of unigenes larger than 190. Table S2 List of gene pairs with Ka/Ks larger than one.
