# Supplementary material for: Molecular Characterization of Donacia provosti (Coleoptera: Chrysomelidae) Larval Transcriptome by De Novo Assembly to Discover Genes Associated with Underwater Environmental Adaptations
Source: Insects. 2021 Mar 25;12(4):281. doi: 10.3390/insects12040281 (PMC8064349; doi:10.3390/insects12040281)
Supplement: Supplementary file 1 [file insects-12-00281-s001.pdf]

Supplementary Files:

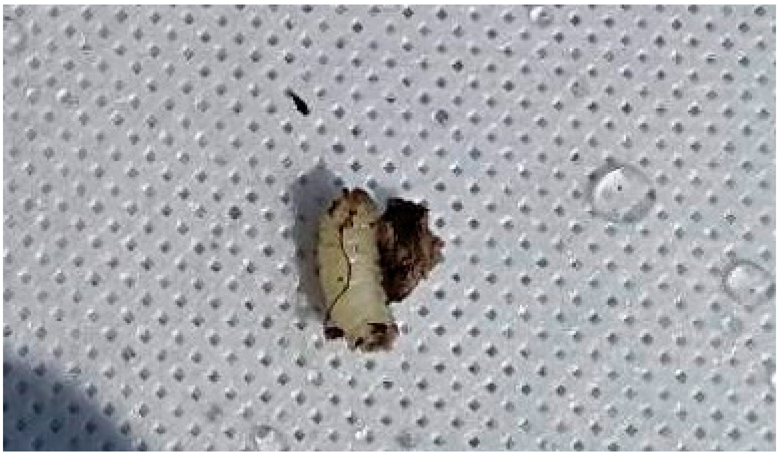

Figure S1 *Donacia provosti* larvae.

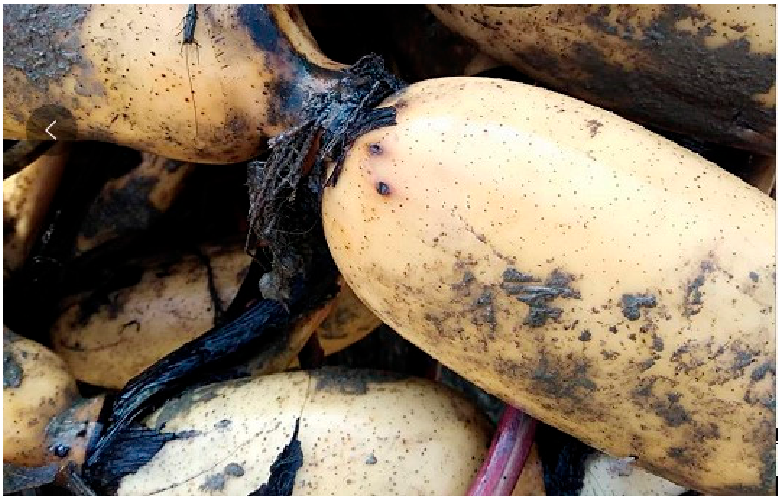

Figure S2 *Donacia provosti* damage characteristics.

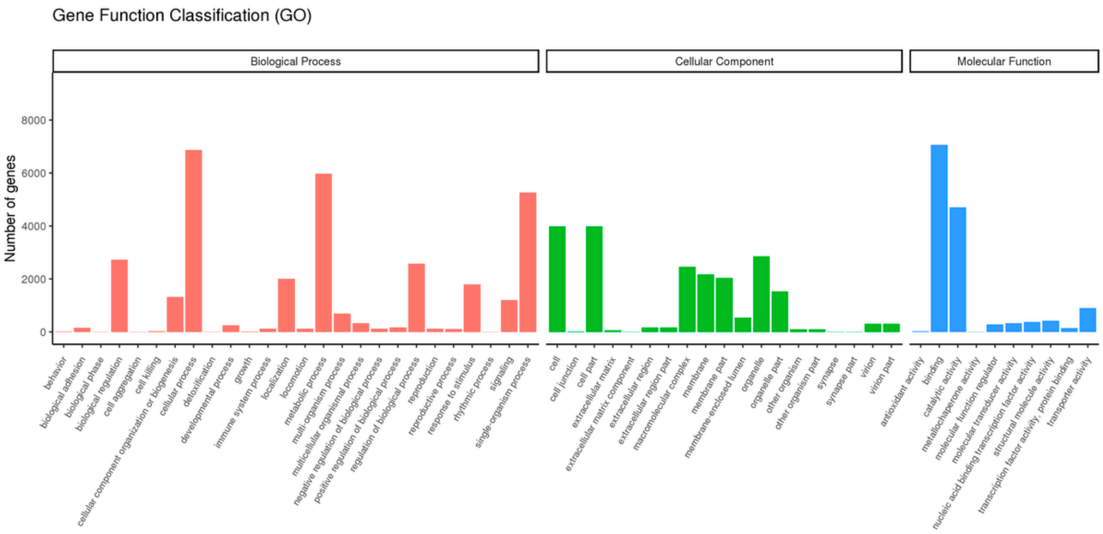

Figure S3. GO Classification of *Donacia provosti* unigenes according to the categories of Biological process, Molecular function, and Cellular component.

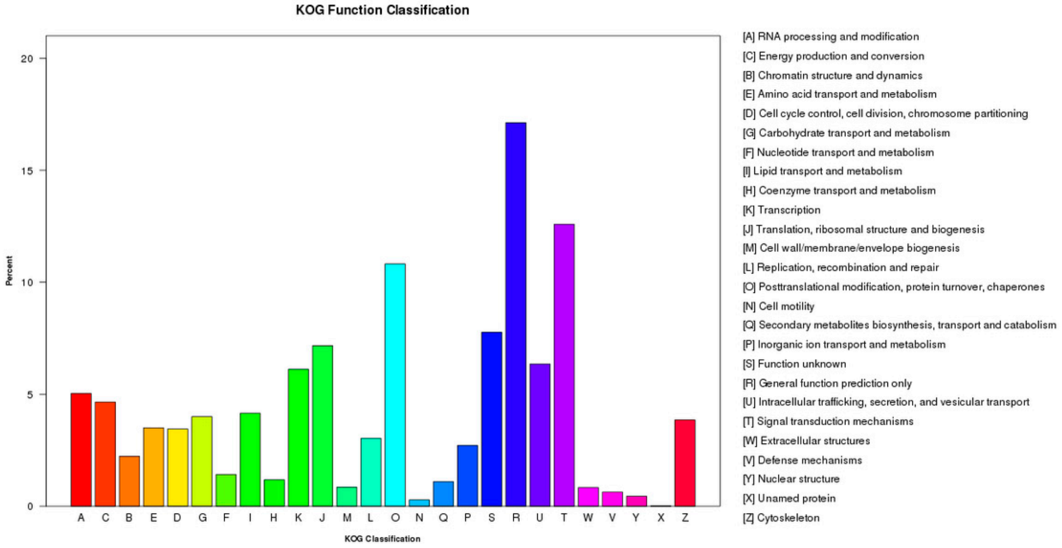

Figure S4. KOG annotations of *Donacia provosti* predicted proteins. A total of 6,678 predicted proteins has a KOG classification among the 26 categories.

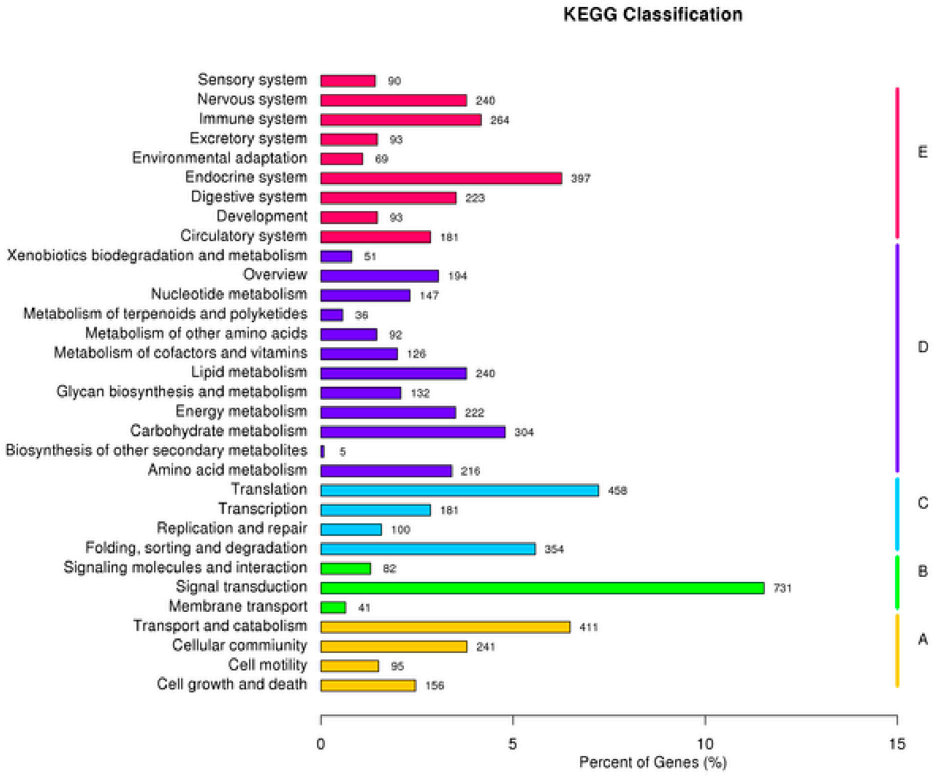

Figure S5. KEGG annotation of *Donacia provosti* predicted proteins. A Cellular Processes, B Environmental Information Processing, C Genetic Information Processing, D Metabolism, E Organismal Systems.

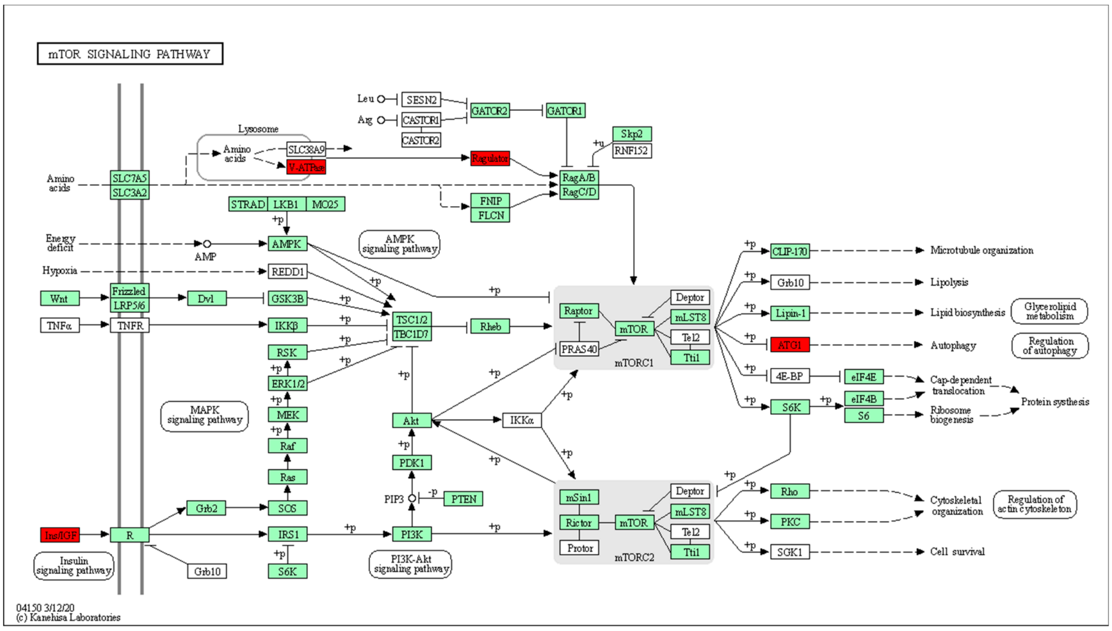

Figure S6 mTOR signaling pathway.

Table S1 List of species with number of unigenes larger than 190

Species	Number of unigenes
Anoplophora glabripennis	6,562
Leptinotarsa decemlineata	3,211
Tribolium castaneum	955
Onthophagus taurus	489
Aethina tumida	381
Dendroctonus ponderosae	337
Myzus persicae	261
Cryptotermes secundus	226
Acyrthosiphon pisum	192

Table S2 List of gene pairs with Ka/Ks larger than one

Gene pair ID		Ka/Ks	Description
Donacia provosti	*Leptinotarsa decemlineata*		
Cluster-865.1	rna-XM_023170247.1	2.92629	PREDICTED: homeotic protein spalt-major isoform X5 [Tribolium castaneum]
Cluster-2724.11667	rna-XM_023160785.1	2.10042	ubiquitin-conjugating enzyme E2 L3 [Leptinotarsa decemlineata]
Cluster-4953.0	rna-XM_023163868.1	1.9729	GTP-binding nuclear protein Ran [Orbicella faveolata]
Cluster-2724.4879	rna-XM_023163408.1	1.95824	dynactin subunit 3 [Anoplophora glabripennis]
Cluster-2724.19997	rna-XM_023169285.1	1.86627	transmembrane protein 185B [Anoplophora glabripennis]
Cluster-2724.10463	rna-XM_023164662.1	1.86161	TIMELESS-interacting protein [Anoplophora glabripennis]
Cluster-2724.14159	rna-XM_023159393.1	1.8027	UDP-GlcNAc:betaGal beta-1,3-N-acetylglucosaminyltransferase-like protein 1 isoform X1 [Leptinotarsa decemlineata]
Cluster-2724.15400	rna-XM_023158726.1	1.75495	ER membrane protein complex subunit 2-like [Anoplophora glabripennis]
Cluster-2724.17421	rna-XM_023165804.1	1.73552	small nuclear ribonucleoprotein F [Anoplophora glabripennis]
Cluster-2724.12272	rna-XM_023173748.1	1.67871	aldose reductase-like [Leptinotarsa decemlineata]
Cluster-2724.4629	rna-XM_023172695.1	1.62534	palmitoyltransferase ZDHHC3 [Anoplophora glabripennis]
Cluster-2724.5357	rna-XM_023157511.1	1.62374	LIRP-like [Anoplophora glabripennis]
Cluster-2724.6055	rna-XM_023160810.1	1.60735	twinkle protein, mitochondrial [Leptinotarsa decemlineata]
Cluster-2724.8746	rna-XM_023166297.1	1.60475	probable ATP-dependent RNA helicase DDX46 [Anoplophora glabripennis]
Cluster-2724.4458	rna-XM_023159903.1	1.59358	PREDICTED: uncharacterized protein LOC660933 [Tribolium castaneum]
Cluster-2724.17411	rna-XM_023158176.1	1.57414	charged multivesicular body protein 1b [Leptinotarsa decemlineata]
Cluster-2724.8472	rna-XM_023174304.1	1.57149	translation machinery-associated protein 16 homolog [Leptinotarsa decemlineata]
Cluster-2724.9697	rna-XM_023169368.1	1.53299	serine..threonine-protein kinase ULK2 isoform X2 [Anoplophora glabripennis]
Cluster-2724.7689	rna-XM_023172044.1	1.48623	DNA replication complex GINS protein PSF2 [Anoplophora glabripennis]
Cluster-2724.15777	rna-XM_023164962.1	1.46514	adrenodoxin-like protein, mitochondrial [Anoplophora glabripennis]
Cluster-2724.7913	rna-XM_023165526.1	1.46453	tetratricopeptide repeat protein 7B [Anoplophora glabripennis]
Cluster-2724.19469	rna-XM_023156727.1	1.46433	probable splicing factor 3B subunit 5 [Anoplophora glabripennis]
Cluster-2724.1072	rna-XM_023171405.1	1.44307	UDP-glucuronosyltransferase 2B31 [Anoplophora glabripennis]
Cluster-2724.2670	rna-XM_023159710.1	1.43624	zinc finger MYND domain-containing protein 11 [Anoplophora glabripennis]
Cluster-2724.17677	rna-XM_023168468.1	1.42807	39S ribosomal protein L46, mitochondrial isoform X1 [Anoplophora glabripennis]
Cluster-2724.11966	rna-XM_023170166.1	1.41905	protein seele isoform X1 [Leptinotarsa decemlineata]
Cluster-2724.9696	rna-XM_023166069.1	1.41138	uncharacterized protein LOC111510180 [Leptinotarsa decemlineata]

Cluster-2724.8608	rna-XM_023164659.1	1.40224	NSFL1 cofactor p47 [Anoplophora glabripennis]
Cluster-2724.10512	rna-XM_023166186.1	1.39254	uncharacterized protein LOC108905236 [Anoplophora glabripennis]
Cluster-2724.1940	rna-XM_023157652.1	1.39058	zinc finger protein ZFP69 isoform X1 [Anoplophora glabripennis]
Cluster-2724.6191	rna-XM_023165242.1	1.36932	PREDICTED: 39S ribosomal protein L13, mitochondrial [Dendroctonus ponderosae]
Cluster-2724.19937	rna-XM_023156725.1	1.36599	N-acetyltransferase ESCO2 [Anoplophora glabripennis]
Cluster-2724.12587	rna-XM_023166835.1	1.36045	V-type proton ATPase subunit d [Leptinotarsa decemlineata]
Cluster-2724.18399	rna-XM_023160042.1	1.34297	COMM domain-containing protein 4 [Anoplophora glabripennis]
Cluster-2724.11150	rna-XM_023160109.1	1.30929	interleukin enhancer-binding factor 2 homolog [Anoplophora glabripennis]
Cluster-2724.1113	rna-XM_023166665.1	1.29696	succinate dehydrogenase assembly factor 4, mitochondrial [Anoplophora glabripennis]
Cluster-2724.1793	rna-XM_023160326.1	1.29162	uncharacterized protein LOC108910374 isoform X1 [Anoplophora glabripennis]
Cluster-2724.9240	rna-XM_023160179.1	1.28195	ubiquinone biosynthesis monooxygenase COQ6, mitochondrial [Anoplophora glabripennis]
Cluster-2724.6166	rna-XM_023158017.1	1.27882	kinesin-like protein KIF3A [Anoplophora glabripennis]
Cluster-2724.7753	rna-XM_023167874.1	1.2697	uncharacterized protein LOC108916674 [Anoplophora glabripennis]
Cluster-2724.4701	rna-XM_023160521.1	1.25986	Ankyrin repeat containing protein [Acanthamoeba castellanii str. Neff]
Cluster-2724.12153	rna-XM_023158112.1	1.25019	protein yippee-like 5 [Anoplophora glabripennis]
Cluster-2724.12451	rna-XM_023162019.1	1.22864	vacuolar protein sorting-associated protein 72 homolog [Anoplophora glabripennis]
Cluster-2724.13315	rna-XM_023167910.1	1.22435	PREDICTED: cuticular protein analogous to peritrophins 1-H isoform X1 [Tribolium castaneum]
Cluster-2724.18632	rna-XM_023162138.1	1.21982	protein farnesyltransferase..geranylgeranyltransferase type-1 subunit alpha [Anoplophora glabripennis]
Cluster-2724.19239	rna-XM_023168057.1	1.20714	uncharacterized protein LOC108907222 [Anoplophora glabripennis]
Cluster-2724.3186	rna-XM_023158032.1	1.20489	mitochondrial import receptor subunit TOM70 [Anoplophora glabripennis]
Cluster-2724.10149	rna-XM_023165266.1	1.18949	uncharacterized protein LOC111691779 [Anoplophora glabripennis]
Cluster-2724.12806	rna-XM_023161499.1	1.18812	m7GpppN-mRNA hydrolase [Leptinotarsa decemlineata]
Cluster-2724.11425	rna-XM_023157388.1	1.18338	40S ribosomal protein S19 [Anoplophora glabripennis]
Cluster-2724.5474	rna-XM_023156520.1	1.18073	charged multivesicular body protein 2a [Leptinotarsa decemlineata]
Cluster-2724.5301	rna-XM_023157318.1	1.1734	uncharacterized protein C7orf50 [Anoplophora glabripennis]
Cluster-2724.9129	rna-XM_023166749.1	1.16672	peroxiredoxin-5, mitochondrial [Anoplophora glabripennis]
Cluster-2724.14456	rna-XM_023171909.1	1.16475	protein-serine O-palmitoleoyltransferase porcupine [Anoplophora glabripennis]
Cluster-2724.12495	rna-XM_023158233.1	1.1603	serine..arginine-rich splicing factor 7-like isoform X2 [Anoplophora glabripennis]
Cluster-2724.9679	rna-XM_023174634.1	1.15916	uncharacterized protein LOC108906306 [Anoplophora glabripennis]
Cluster-2724.7527	rna-XM_023164602.1	1.158	DNA-directed RNA polymerases I, II, and III subunit RPABC4 [Anoplophora glabripennis]

Cluster-2724.6124	rna-XM_023168881.1	1.15754	zinc finger CCHC domain-containing protein 4 [Anoplophora glabripennis]
Cluster-2724.19728	rna-XM_023168364.1	1.14417	DNA..RNA-binding protein KIN17 [Anoplophora glabripennis]
Cluster-2724.7533	rna-XM_023167827.1	1.14396	uncharacterized protein LOC111511807 [Leptinotarsa decemlineata]
Cluster-2724.6156	rna-XM_023165162.1	1.13637	trafficking protein particle complex subunit 6B [Anoplophora glabripennis]
Cluster-2724.11583	rna-XM_023169201.1	1.09581	26S proteasome non-ATPase regulatory subunit 12 [Anoplophora glabripennis]
Cluster-2724.2029	rna-XM_023168612.1	1.09568	transient receptor potential channel pyrexia-like isoform X1 [Leptinotarsa decemlineata]
Cluster-2724.2735	rna-XM_023158705.1	1.08433	protein PRRC1 [Anoplophora glabripennis]
Cluster-2724.8588	rna-XM_023168706.1	1.08067	probable phospholipid hydroperoxide glutathione peroxidase [Leptinotarsa decemlineata]
Cluster-2724.17642	rna-XM_023174187.1	1.07675	26S proteasome non-ATPase regulatory subunit 13 [Leptinotarsa decemlineata]
Cluster-2724.6906	rna-XM_023167847.1	1.07344	ragulator complex protein LAMTOR4 homolog [Anoplophora glabripennis]
Cluster-2724.9880	rna-XM_023156878.1	1.06693	SWI..SNF-related matrix-associated actin-dependent regulator of chromatin subfamily B member 1 [Anoplophora glabripennis]
Cluster-2724.5990	rna-XM_023161016.1	1.06682	mitochondrial import receptor subunit TOM22 homolog [Anoplophora glabripennis]
Cluster-2724.12324	rna-XM_023159072.1	1.06484	5'-3' exoribonuclease 2 homolog [Anoplophora glabripennis]
Cluster-2724.9784	rna-XM_023163758.1	1.06456	probable peptidyl-tRNA hydrolase 2 [Anoplophora glabripennis]
Cluster-2724.8564	rna-XM_023169323.1	1.06119	golgin subfamily A member 7 [Leptinotarsa decemlineata]
Cluster-2724.18993	rna-XM_023163520.1	1.05185	uncharacterized protein LOC111508101 isoform X1 [Leptinotarsa decemlineata]
Cluster-2724.3964	rna-XM_023160252.1	1.0497	argininosuccinate lyase [Anoplophora glabripennis]
Cluster-2724.13509	rna-XM_023156704.1	1.04818	UDP-glucuronosyltransferase 2B15 [Anoplophora glabripennis]
Cluster-2724.16264	rna-XM_023162708.1	1.04651	LOW QUALITY PROTEIN: selenoprotein F [Anoplophora glabripennis]
Cluster-2724.8063	rna-XM_023162057.1	1.04607	nucleolar complex protein 2 homolog [Anoplophora glabripennis]
Cluster-2724.15826	rna-XM_023164542.1	1.03893	enhancer of split mbeta protein-like [Anoplophora glabripennis]
Cluster-2724.7520	rna-XM_023156778.1	1.03882	Protein couch potato-like Protein [Tribolium castaneum]
Cluster-2724.11785	rna-XM_023168031.1	1.03666	translationally-controlled tumor protein homolog [Leptinotarsa decemlineata]
Cluster-2724.7271	rna-XM_023173705.1	1.03623	Cytochrome b5-like Protein [Tribolium castaneum]
Cluster-2724.7274	rna-XM_023173847.1	1.03181	leucine-rich repeat protein SHOC-2 isoform X1 [Anoplophora glabripennis]
Cluster-2724.10504	rna-XM_023168801.1	1.02782	60S ribosomal protein L29 [Anoplophora glabripennis]
Cluster-2724.8398	rna-XM_023164356.1	1.02736	28S ribosomal protein S9, mitochondrial [Leptinotarsa decemlineata]
Cluster-2724.4720	rna-XM_023160351.1	1.02032	probable RNA-binding protein 18 [Anoplophora glabripennis]
Cluster-2724.8044	rna-XM_023169701.1	1.01858	solute carrier family 15 member 1 [Anoplophora glabripennis]

Cluster-2724.10849	rna-XM_023172726.1	1.01646	V-type proton ATPase subunit E [Leptinotarsa decemlineata]
Cluster-2724.9463	rna-XM_023160460.1	1.01544	WD repeat-containing and planar cell polarity effector protein fritz [Anoplophora glabripennis]
Cluster-2724.17243	rna-XM_023173708.1	1.01382	WD repeat-containing protein 5 [Anoplophora glabripennis]
Cluster-2724.17379	rna-XM_023172260.1	1.01331	charged multivesicular body protein 2b-B [Anoplophora glabripennis]
Cluster-2724.11547	rna-XM_023166664.1	1.0108	sperm-associated antigen 7 [Anoplophora glabripennis]
Cluster-2724.5878	rna-XM_023160638.1	1.0072	AN1-type zinc finger protein 1-like [Leptinotarsa decemlineata]
Cluster-2724.12953	rna-XM_023173478.1	1.00498	asparagine synthetase [glutamine-hydrolyzing] [Anoplophora glabripennis]
